# Pichia kurtzmaniana f.a. sp. nov., with the transfer of eight Candida species to Pichia

**DOI:** 10.1099/ijsem.0.006306

**Published:** 2024-03-27

**Authors:** Hai-Yan Zhu, Liang-Chen Guo, Shuang Hu, Yu-Hua Wei, Feng-Li Hui, Xin-Zhan Liu, Feng-Yan Bai

**Affiliations:** 1 State Key Laboratory of Mycology, Institute of Microbiology, Chinese Academy of Sciences, Beijing 100101, PR China; 2 College of Life Sciences, University of Chinese Academy of Sciences, Beijing 100049, PR China; 3 School of Life Science and Agricultural Engineering, Nanyang Normal University, Nanyang 473061, PR China

**Keywords:** *Candida*, new combinations, *Pichia*, *Pichia kurtzmaniana *sp. nov.

## Abstract

Three yeast strains belonging to the ascomycetous yeast genus *Pichia* were isolated from two soil samples from Yunnan and Guizhou provinces and a marine water sample from Liaoning province, PR China. Phylogenetic analyses based on the sequences of the D1/D2 domains of the large subunit(LSU) rRNA gene and the internal transcribed spacer (ITS) region indicate that these three strains, together with 12 additional strains isolated from various substrates collected in different regions or countries of the world, represent a novel species of the genus *Pichia*, for which the name *Pichia kurtzmaniana* sp. nov. (holotype: strain CGMCC 2.7213) is proposed. The novel species differs from its close relatives *Candida californica* by eight (1.5 %) and 26 (11.1 %) mismatches in the D1/D2 domains and the ITS region, respectively; and from *Pichia chibodasensis* by 11 (2.1 %) and 20 (8.7 %) mismatches in the D1/D2 domains and the ITS region, respectively. In addition, eight *Candida* species which belong to the *Pichia* clade are transferred to the genus *Pichia*, resulting in the proposal of the following new combinations: *Pichia cabralensis* comb. nov., *Pichia californica* comb. nov., *Pichia ethanolica* comb. nov., *Pichia inconspicua* comb. nov., *Pichia phayaonensis* comb. nov., *Pichia pseudolambica* comb. nov., *Pichia rugopelliculosa* comb. nov., and *Pichia thaimueangensis* comb. nov.

## Introduction

The genus *Pichia* (*Ascomycota*, *Saccharomycotina*, *Pichiomycetes*, *Pichiales*) was established by Hansen in 1904 with *Pichia membranifaciens* as the type species [1,2]. It is characterized by multilateral budding, occasionally presence of pseudohyphae but not hyphae, and forming ascospores that may be hat-shaped, hemi-spheroidal, or spherical with or without a ledge [3]. Almost all the species in this genus can ferment glucose, but do not assimilate nitrate [3]. Based on the phylogenetic analysis of the large and small subunit (LSU and SSU) rRNA genes and the translation elongation factor-1a (*tef-1a*) gene, Kurtzman *et al*. examined phylogenetic relationships among species of the genera *Pichia*, *Issatchenkia* and *Williopsis*, and redefined the genus *Pichia* [3]. Consequently, Kurtzman accepted 20 ascosporogenous species in the genus *Pichia* in the fifth edition of *The Yeasts, a Taxonomic Study* [2]. Since then, several *Pichia* species have been described, including *Pichia chibodasensis* isolated from decayed wood and soil in Indonesia [4], *Pichia dushanensis* from the gut of insect larvae in PR China [5], *Pichia bruneiensis* from flowers in Borneo [6], *Pichia insulana* from necrotic tissue of columnar cacti in Caribbean [7], and *Pichia nanzhaoensis* and *Pichia paraexigua* from rotting wood in PR China [8]. Additionally, eight *Candida* species are known to be members of the *Pichia* clade [8].

During a survey of yeast diversity from the intertidal zones along the Chinese coastline and minority areas of PR China, three strains isolated from soil collected in Yunnan and Guizhou provinces and marine water collected in Liaoning province were found to represent a novel species in the genus *Pichia* based on sequence comparisons of the D1/D2 domains of the large subunit (LSU) rRNA gene and the internal transcribed spacer (ITS) region. We found that 12 additional strains isolated from different substrates collected from different regions of the world also belong to the new *Pichia* species together with our three Chinese strains. In addition to the description of the new species, we transfer eight *Candida* species that are included in the *Pichia* clade to *Pichia* according to the International Code of Nomenclature for algae, fungi, and plants (Shenzhen Code) [9], which requests phylogenetically related anamorphic or teleomorphic species to be assigned to the same genus.

## Samples collection and yeast isolation

Samples of marine water were collected from intertidal zones in Liaoning province, northeast China in October 2020. Yeast isolation from marine water was performed by filtrate collection and inoculation method described by Zhu *et al*. [10]. Samples of soil were collected from Yunnan and Guizhou provinces in February and November, 2022, respectively. Yeast isolation from soil samples was done by an enrichment method with minor modifications described by Bai *et al*. and Wang *et al*. [11,12]. Specifically, 2 g of each sample were placed into 10 ml YM broth (w/v, 1 % glucose, 0.5 % peptone, 0.3 % yeast extract and 0.3 % malt extract) supplemented with 7 % ethanol, 200 µg ml^−1^ chloramphenicol, and 1 ml of 1 M HCl per litre in a 15 ml sterile centrifuge tube and then incubated at 25 °C for 1 week. Then 100 µl enrichment culture and appropriate decimal dilutions were spread on YM agar plates supplemented with 200 µg ml^−1^ chloramphenicol and then incubated at 25 °C for 3–4 days. Different yeast morphotypes were picked, and pure colonies were obtained after at least two streak plate procedures and then stored in 25 % glycerol at −80 °C.

## Phenotypic characterization

Morphological characteristics and physiological and biochemical properties were examined according to standard methods described by Kurtzman *et al*. [13]. The assimilation of carbon and nitrogen compounds was conducted in liquid media. The potential sexual cycles of strains representing new species were investigated using corn meal agar (CMA; w/v, 2.5 % corn starch and 2 % agar), potato dextrose agar (PDA; w/v, 20% potato infusion, 2% glucose, and 2% agar), YM agar and V8 agar (w/v, 10 % V8 juice and 2 % agar). A loopful of cells of each test strain was inoculated separately or mixed on agar plates and incubated at 25 °C for up to 2 months and examined periodically.

## Molecular phylogenetic analysis

DNA of yeast cells was extracted according to the method described by Wang and Bai [14]. The fragment covering the ITS region and D1/D2 domains was amplified and sequenced using the methods described previously [12]. Sequence alignment was performed using mafft version 7 [15] and manually improved where it was necessary using mega version 7 [16]. Phylogenetic analyses based on single D1/D2 or ITS sequences was performed based on the evolutionary distance data calculated from Kimura’s two parameter model using the neighbour-joining algorithm executed in mega version 7 [16,18]. Maximum-likelihood phylogenetic analysis based on the concatenated D1/D2 and ITS sequences was performed using the best-fit model GTR+I+G determined in mega version 7 [16]. Bootstrap analyses were performed from 1000 random re-samplings [19].

## Molecular phylogenetic analyses

Two strains, W2E-1 and YPD123-1, isolated from soil samples collected in Yunnan and Guizhou provinces, respectively, southwest China, and strain 200 A-5, isolated from marine water collected in an intertidal zone in Liaoning province, northeast China (Table 1), possessed identical ITS sequences and similar (only one base mismatch) D1/D2 sequences, suggesting they are conspecific. These yeast strains were primarily identified using blast searches through GenBank with their ITS and D1/D2 sequences as queries. The D1/D2 sequence blast result indicated that 10 strains, which were isolated previously from different substrates collected in different regions or countries, showed identical or similar D1/D2 sequences to the three new Chinese strains. These strains were previously identified as *Candida californica*, *Candida ethanolica*, *Pichia scaptomyzae*, *Candida* sp. or *Pichia* sp. (Table 1 and Fig. S1, available in the online version of this article). The ITS sequences are available for three (Af140S-3–1, UFMG-CM-Y6900, and NYNU 13710) of these 10 strains. The phylogenetic analysis based on the ITS sequences showed that the three Chinese strains formed a distinct clade together with strains Af140S-3–1, UFMG-CM-Y6900, NYNU 13710, and two additional strains isolated from USA which were previously identified as *Pichia scaptomyzae* (yHRVM277) and *Candida californica* (yHKB455) (Fig. S2) [20]. The strains in this clade possess identical ITS sequences. The sequence comparisons mentioned above suggested that the three strains isolated in this study and the 12 strains isolated previously by other researchers are most likely conspecific.

**Table 1.** The yeast species and strains employed in this study The strains in bold were isolated in this study. Type strains are denoted with the superscript ‘T’. The sequences extracted from the released genome sequences of the strains concerned are marked with an asterisk.

Current name	Previous identification	Strain	Source	Origin	Accession no.	
					ITS	D1/D2
*Pichia barkeri*		NRRL Y-17350^T^	Plant	Jamaica	NR_153283	EF550247
*Pichia bovicola*		DMKU-MP6-4^T^	Small-intestine of cattle	Thailand	MZ841616	MZ322503
*Pichia bruneiensis*		CBS 12611^T^	Flowers	Borneo	NR173357	NG075177
*Pichia cabralensis* comb. nov.	*Candida cabralensis*	CBS 11679^T^	Food	Spain	KY102010	FJ755462
*Pichia cactophila*		NRRL Y-10963^T^	Organ pipe cactus	Mexico	NR_138243	EF550241
*Pichia californica* comb. nov.	*Candida californica*	NRRL Y-27254^T^	Fruit	USA	NR_153280	EF550230
*Pichia cecembensis*		NRRL Y-27985^T^	Rotten papaya fruit	India	AM233511	AM159112
*Pichia cephalocereana*		NRRL Y-17225^T^	Rot of columnar cactus	West Indies	NR153285	EF550250
*Pichia chibodasensis*		NBRC 111569^T^	Soil	Indonesia	LC126435	LC126429
*Pichia deserticola*		NRRL Y-12918^T^	Prickly pear cactus	USA	NR_077085	EF550226
*Pichia dushanensis*		NYNU 14658^T^	Gut of insect larvae	China	KM272245	KM272244
*Pichia eremophila*		NRRL Y-17224^T^	Plant	USA	NR_153287	EF550249
*Pichia ethanolica* comb. nov.	*Candida ethanolica*	NRRL Y-12615^T^	Industrial fodder yeast	Czechoslovakia	NR077165	EF550225
*Pichia exigua*		NRRL Y-10920^T^	Insect	USA	NR_153288	EF550237
*Pichia fermentans*		NRRL Y-1619^T^	Buttermilk	Netherlands	NR_130688	EF550234
*Pichia gijzeniarum*		CBS 15024^T^	Soil	Netherlands	MG986490	MG986495
*Pichia heedii*		CBS 6930^T^	Cactus	Mexico	KY104553	KY108818
*Pichia inconspicua* comb. nov.	*Candida inconspicua*	NRRL Y-2029^T^	Sputum	Netherlands	Not available	EF550240
*Pichia insulana*		CBS 11169^T^	Rotten cactus *Cereus repandus*	Caribbean	EU747339	KM252834
*Pichia jaroonii*		CBS 10930^T^	Forest soil	Thailand	Not available	AB436766
*Pichia kluyveri*		NRRL Y-11519^T^	Olives	USA	NR_138210	EF550251
*Pichia kudriavzevii*		NRRL Y-5396^T^	Fruit juice	Russia	MW284497	U76347
***Pichia kurtzmaniana*** **f.a.sp. nov.**		**W2E-1=CGMCC2.7214=JCM36239**	**Soil**	**Yunnan, PR China**	**OR258061**	**OR258061**
		**YPD123−1=CGMCC 2.7213^T^=JCM 36238**	**Soil**	**Guizhou, PR China**	**OR258060**	**OR258060**
		**200 A-5 = CGMCC 2.10117**	**Marine water**	**Liaoning, PR China**	**OR258057**	**OR258059**
	*Candida californica*	yHKB455	Duff	USA	OK050907	Not available
	*Candida californica*	A1MYC-1	Mycangium	PR China	Not available	ON838569
	*Candida ethanolica*	NCYC 3463	Spoiled strawberry soft drink	United Kingdom	Not available	HF547282
	*Candida* sp.	JW01-7-11-2-1-y2	Gut of scolytid beetle	Panama	Not available	AY242330
	*Candida* sp.	JCM 28228	Black viscous substances	Japan	Not available	LC134046
	*Candida* sp.	CBS 6394	Unknown	Unknown	Not available	AY551000
	*Pichia scaptomyzae*	yHRVM277	Sand	USA	OK052400	Not available
	*Pichia scaptomyzae*	NCYC 3726	Spoiled strawberry soft drink	United Kingdom	Not available	HF547284
	*Pichia* sp.	NYNU 13710	Unknown	PR China	KF690380	KF690367
	*Pichia* sp.	Af140S-3–1	Fermented sap of *Quercus Serrata*	Japan	LC661447	LC661415
	*Pichia* sp.	UFMG-CM-Y6900	Soil	Brazil	OM480681	OM480681
	*Pichia* sp.	UWO(PS)85–301.3	Flux of *Prosopis juliflora*	USA	Not available	AF530614
*Pichia manshurica*		NRRL Y-17349^T^	Faeces	Japan	NR_138211	EF550223
*Pichia membranifaciens*		NRRL Y-2026^T^	Unknown	Unknown	NR_111195	EF550227
*Pichia nakasei*		NRRL Y-7686^T^	Apple must	Chile	KY104634	EF550248
*Pichia nanzhaoensis*		NYNU 178136^T^	Rotten wood	PR China	MG255719	MG255700
*Pichia norvegensis*		NRRL Y-7687^T^	Vagina of a pregnant woman	United Kingdom	KY104640	EF550239
*Pichia occidentalis*		CBS 5459^T^	Unknown	Unknown	GCA_003705455*	U76348
*Pichia paraexigua*		NYNU 178136^T^	Rotten wood	PR China	MG255719	MG255700
*Pichia phayaonensis* comb. nov.	*Candida phayaonensis*	CBS 12319^T^	Soil	Thailand	KY102331	KY106689
*Pichia pseudocactophila*		NRRL Y-17239^T^	Cactus	Mexico	GCA_030580035*	EF550242
*Pichia pseudolambica* comb. nov.	*Candida pseudolambica*	CBS 2063^T^	Silage	United Kingdom	MK394161	U71063
*Pichia rugopelliculosa* comb. nov.	*Candida rugopelliculosa*	CBS 6377^T^	Food	Japan	KY102367	U71069
*Pichia scutulata*		NRRL Y-7663^T^	Plant	USA	KY104645	EF550243
*Pichia sporocuriosa*		NRRL Y-27347^T^	Tree	Malaysia	NR_153293	EF550232
*Pichia terricola*		CBS 2617^T^	Soil	South Africa	NR_153294	KY108920
*Pichia thaimueangensis* comb. nov.	*Candida thaimueangensis*	CBS 10360^T^	Water	Thailand	KY102439	AB264009
*Kregervanrija fluxuum*		CBS 2287=NRRL YB-4273^T^	Sap of black oak	USA	EF550268	AY923249
*Martiniozyma abietophila*		CBS 5366=NRRL Y-11514^T^	Sap of red fir	USA	NG066352	NR_161000

**Fig. 1.**
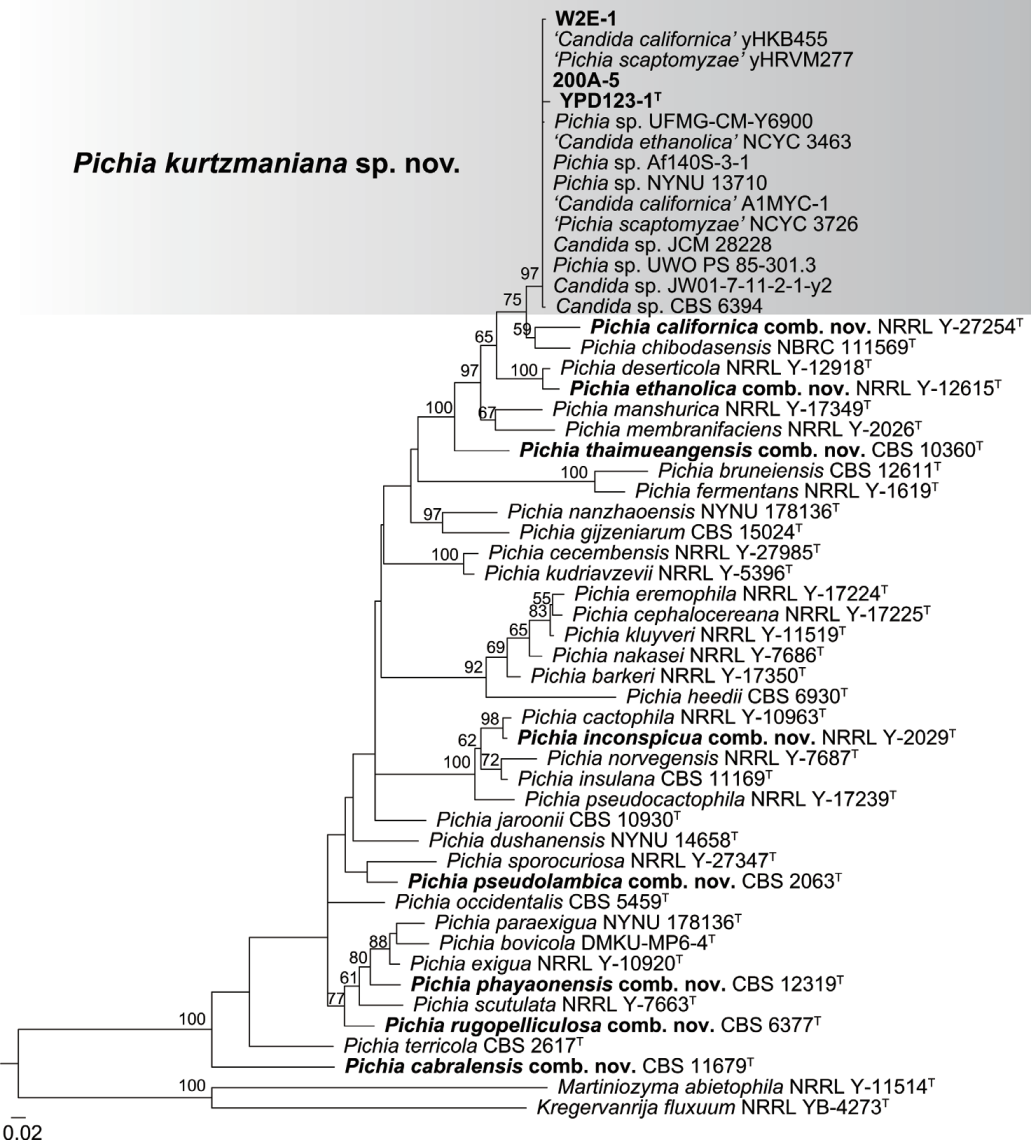
Maximum-likelihood phylogenetic tree based on the concatenated D1/D2 and ITS sequences showing the phylogenetic position of *Pichia kurtzmaniana* sp. nov. Bootstrap percentages >50 % from 1000 replicates are shown. *Kregervanrija fluxuum* and *Martiniozyma abietophila* are used as the outgroup. Type strains are denoted with the superscript ‘T’. Strains isolated in this study and the new combinations are marked in bold. Bar, 0.02 substitutions per nucleotide position.

The phylogenetic analyses based on the concatenated D1/D2 and ITS sequences confirmed the affinity of the new group represented by strain YPD123-1 to the genus *Pichia* with high bootstrap support (Fig. 1). The YPD123-1 group was closely related to *Candida californica* (=*Pichia californica* comb. nov.) and *Pichia chibodasensis* in the trees reconstructed from the D1/D2 (Fig. S1) and the combined D1/D2 and ITS sequences (Fig. 1). The YPD123-1 group exhibited eight (1.5 %) and 11 (2.1 %) base differences from the type strains of *Candida californica* and *Pichia chibodasensis*, respectively, and 4 % or more mismatches from the other described species of the genus *Pichia* in the D1/D2 domains. This group exhibited 26 (11.1 %) and 20 (8.7 %) base differences from the type strains of *Candida californica* and *Pichia chibodasensis*, respectively, in the ITS region. The previous names *Candida californica*, *Candida ethanolica,* and *Pichia scaptomyzae *of some strains in the YPD123-1 group (Table 1 and Fig. 1) were obviously misidentification. Ueda-Nishimura and Mikata [21] has proved that *Pichia scaptomyzae* is a synonym of *Pichia membranifaciens*, the type species of the genus [21]. The YPD123-1 group is clearly differentiated from *Pichia membranifaciens*, *Candida californica* (=*Pichia californica* comb. nov.), and *Candida ethanolica* (=*Pichia ethanolica* comb. nov.) phylogenetically (Figs 1, S1 and S2). These results indicated that the YPD123-1 group represents a novel species. Though the new species represented by the YPD123-1 group is closely related to the anamorphic species *Candida californica* and a sexual state has not been observed in any of the strains in this group, according to the Shenzhen Code [9], we assign the new species to the genus *Pichia* and propose the name *Pichia kurtzmaniana* sp. nov. for it. Likewise, we transfer *Candida californica* and seven additional *Candida* species, that are clustered in the *Pichia* clade with strong bootstrap support in the phylogenetic trees (Figs 1, S1 and S2) to the genus *Pichia*.

## Phenotypical characteristics and ecology

Strains W2E-1, YPD123-1 and 200 A-5 of *Pichia kurtzmaniana* sp. nov. form smooth, light-cream coloured, and butyrous colonies on YM agar after growth at 25 °C for 3 days. Physiologically, the new species differed from its close relatives *Pichia californica* comb. nov. and *Pichia chibodasensis* by its abilities to grow at 37 °C and on the agar containing 50 % glucose (Table 2). Salient physiological characteris that differentiate the new species from other closely related species are shown in Table 2.

**Table 2.** Salient phenotypic characteristics that differentiate *Pichia kurtzmaniana* sp. nov. from closely related species +, Positive; −, negative; s, slow; w, weak; v, variable.

Species	Growth at/with			Assimilation of d-xylose
	37 °C	50 % glucose	Vitamin-free	
*P. kurtzmaniana* sp. nov.	+	+	+	+
*P. californica* comb. nov.	−	−	w	v
*P. chibodasensis*	−	−	+	w
*P. deserticola*	+	not available	−	−
*P. ethanolica* comb. nov.	+	−	+	−
*P. manshurica*	+	not available	v	−
*P. membranifaciens*	−	not available	v	v
*P. thaimueangensis* comb. nov.	+	w	−	s

The 15 strains representing *P. kurtzmaniana* sp. nov. are from various sources in different countries, including soil and marine water in PR China, soil in Brazil, deteriorated strawberry soft drinks in the United Kingdom, and the fermentation broth and black viscous substances in Japan, indicating that the novel species is widely distributed in the world.

## Description of *Pichia kurtzmaniana* f.a. sp. nov. H.Y. Zhu, X.Z. Liu and F.Y. Bai

*Pichia kurtzmaniana* (kurtz.man.i.a’na. N.L. fem. adj. *kurtzmaniana*, of Kurtzman, named in honour of C. P. Kurtzman for his great contributions to the systematics of yeasts).

**Fig. 2.**
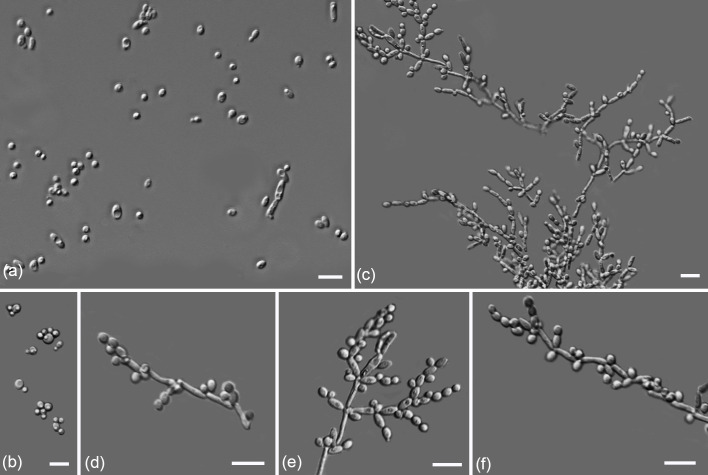
Morphology of *Pichia kurtzmaniana* sp. nov. (strain CGMCC 2.7213^T^). (a−b) Vegetative cells on YM agar at 25 °C for 3 days. (c−f) Pseudohyphae on PDA after 2 weeks at 25 °C. Bars, 10 µm.

Culture characteristics: Cells are ovoid to ellipsoidal (2.6–2.9×2.9–8.8 µm) and occur singly or in pairs (Fig. 2a). Reproduction is by multilateral budding when grown on YM agar at 25 °C (Fig. 2b). On YM agar at 25 °C for 3 days, the colonies are smooth, light-cream in colour, and butyrous. Pseudohyphae are formed after incubation for 2 weeks on PDA (Fig. 2c). Sexual structures were not observed in single- or mixed-strain cultures on CMA, PDA and V8 agar after 2 months of incubation at 25 °C.

Physiological and biochemical characteristics: Glucose is fermented, but galactose, lactose, maltose, sucrose, trehalose and raffinose are not fermented. Glucose, l-sorbose, d-xylose, d-glucosamine, ethanol, glycerol (slow/weak), glucitol (weak), dl-lactic acid, succinic acid (slow/weak), N-acetyl-d-glucosamine and xylitol are assimilated as sole carbon sources. d-galactose, sucrose, maltose, cellobiose, trehalose, lactose, melibiose, raffinose, melezitose, inulin, soluble starch, l-arabinose, d-arabinose, d-ribose, l-rhamnose, methanol, erythritol, ribitol, galactitol, d-mannitol, methyl α-d-glucoside, salicin, d-glucuronic acid, sodium citrate dihydrate, inositol, and hexadecane are not assimilated as sole carbon sources. Ethylamine hydrochloride, cadaverine dihydrochloride, l-lysine, and ammonium sulphate are assimilated as sole nitrogen sources. Sodium nitrite and potassium nitrate are not assimilated as the sole nitrogen sources. Urease activity is negative. Diazonium Blue B reaction is negative. Extracellular amyloid compounds are not produced. Growth in 10 % (w/v) sodium chloride plus 5 % (w/v) glucose medium is positive. Growth on 50 % (w/v) glucose–yeast extract agar and 60 % (w/v) glucose–yeast extract agar is positive. Growth in vitamin-free medium is positive. Weak growth occurs on YM agar at 37 °C, but not at 42 °C.

The holotype, CGMCC 2.7213, was isolated from a soil sample collected from Rongjiang county, Guizhou province, southwest China in November 2022 (original number YPD123-1), and has been deposited in a metabolically inactive state in the China General Microbiological Culture Collection Center (CGMCC), Beijing, PR China. The ex-type culture has been deposited in the Japan Collection of Microorganisms (JCM), Koyadai, Japan, as JCM 36238. The other two Chinese strains of the new species have also been deposited in CGMCC and JCM as CGMCC 2.7214 and JCM 36239 for strain W2E-1; and as CGMCC 2.10117 for strain 200A-5. The GenBank/EMBL/DDBJ accession numbers for the D1/D2 domains and the ITS region sequences of the type strain CGMCC 2.7213 and strain CGMCC 2.7214 (=W2E-1) are OR258060 and OR258061, respectively. The GenBank/EMBL/DDBJ accession numbers for the D1/D2 domains and the ITS region sequences of strain CGMCC 2.10117 (=200A-5) are OR258059 and OR258057, respectively. The Fungal Names number of *Pichia kurtzmaniana* f.a. sp. nov. is FN571639.

## Descripton of ***Pichia cabralensis*** (Flórez, Belloch, Álv.-Martín, Querol and B. Mayo) H.Y. Zhu, L.C. Guo and F.Y. Bai **f.a. comb. nov**.

Fungal Names No.: FN571640

*Basionym*: *Candida**cabralensis* Flórez, Belloch, Álv.-Martín, Querol and B. Mayo, *Int J Syst Evol Microbiol* **60**, 2673 (2010)

## Descripton of *Pichia californica* (Mrak and McClung ex K.W. Anderson and C.E. Skinner) H.Y. Zhu, L.C. Guo and F.Y. Bai f.a. comb. nov.

Fungal Names No.: FN571646

*Basionyms: Cryptococcus californicus* Mrak and McClung ex K.W. Anderson and C.E. Skinner, *Mycologia* **39**, 169 (1947) and *Candida* *californica* (Mrak and McClung ex K.W. Anderson and C.E. Skinner) F.Y. Bai, Z.W. Wu and V. Robert, *FEMS Yeast Res* **6**, 310 (2006)

## Descripton of *Pichia ethanolica* (Rybářová, Štros and Kock.-Krat.) H.Y. Zhu, L.C. Guo and F.Y. Bai f.a. comb. nov.

Fungal Names No.: FN571641

*Basionym*: *Candidaethanolica* Rybářová, Štros and Kock.-Krat., *Z. Allgemeine Mikrobiologie* **20**, 579 (1980)

## Descripton of *Pichia inconspicua* (Lodder and Kreger-van Rij) H.Y. Zhu, L.C. Guo and F.Y. Bai f.a. comb. nov.

Fungal Names No.: FN571647

*Basionyms*: *Torulopsis inconspicua* Lodder & Kreger-van Rij (1952) and *Candida* *inconspicua* (Lodder and Kreger-van Rij) S.A. Mey. and Yarrow, *Int J Syst Bacteriol* **28** 612 (1978)

## Descripton of *Pichia phayaonensis* (Limtong, Nitiyon, Kaewwichian, Jindamorakot, Am-In and Yongmanitchai) H.Y. Zhu, L.C. Guo and F.Y. Bai f.a. comb. nov.

Fungal Names No.: FN571642

*Basionym*: *Candidaphayaonensis* Limtong, Nitiyon, Kaewwichian, Jindamorakot, Am-In and Yongmanitchai, *Int J Syst Evol Microbiol* **62**, 2789 (2012)

## Descripton of *Pichia pseudolambica* (M.T. Smith and Poot) H.Y. Zhu, L.C. Guo and F.Y. Bai f.a. comb. nov.

Fungal Names No.: FN571643

*Basionym*: *Candidapseudolambica* M.T. Smith and Poot, *Stud Mycol* **31 **, 175 (1989)

## Descripton of *Pichia rugopelliculosa* (Nakase) H.Y. Zhu, L.C. Guo and F.Y. Bai f.a. comb. nov.

Fungal Names No.: FN571644

*Basionym*: *Candida rugopelliculosa* Nakase, *J Gen Appl Microbiol Tokyo* **17**, 391 (1971)

## Descripton of* Pichia thaimueangensis* (Limtong, Yongmanitchai, H. Kawasaki and T. Seki) H.Y. Zhu, L.C. Guo and F.Y. Bai f.a. comb. nov.

Fungal Names No.: FN571645

*Basionym*: *Candida**thaimueangensis* Limtong, Yongmanitchai, H. Kawasaki and T. Seki, *Int J Syst Evol Microbiol* **57**, 651 (2007)

## supplementary material

10.1099/ijsem.0.006306 Uncited Fig. S1.
