# Supplementary material for: Pichia kurtzmaniana f.a. sp. nov., with the transfer of eight Candida species to Pichia
Source: Int J Syst Evol Microbiol. 2024 Mar 27;74(3):006306. doi: 10.1099/ijsem.0.006306 (PMC10995725; doi:10.1099/ijsem.0.006306)
Supplement: Uncited Fig. S1. [file ijsem-74-06306-s001.pdf]

Supplementary materials

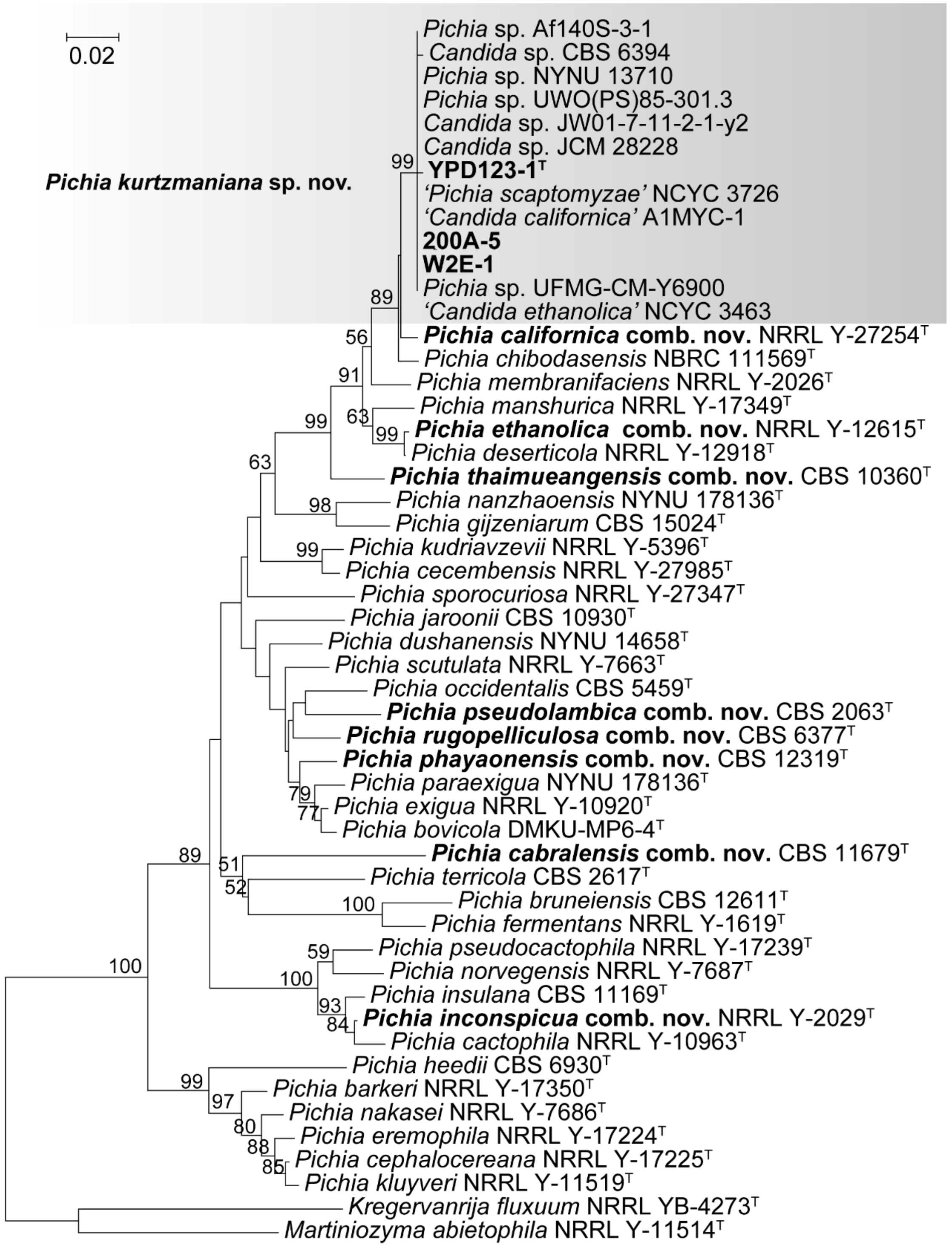

Fig. S1 Neighbor-Joining phylogenetic tree based on the D1/D2 domain sequences showing the phylogenetic position of *Pichia kurtzmaniana* sp. nov. Bootstrap percentages >50% from 1000 replicates are shown. Species *Kregervanrija fluxuum* and *Martiniozyma abietophila* are used as the outgroup. Type strains are denoted with the superscript 'T'. Strains isolated in this study and the new combinations are marked in bold. Bar, 0.02 substitutions per nucleotide position.

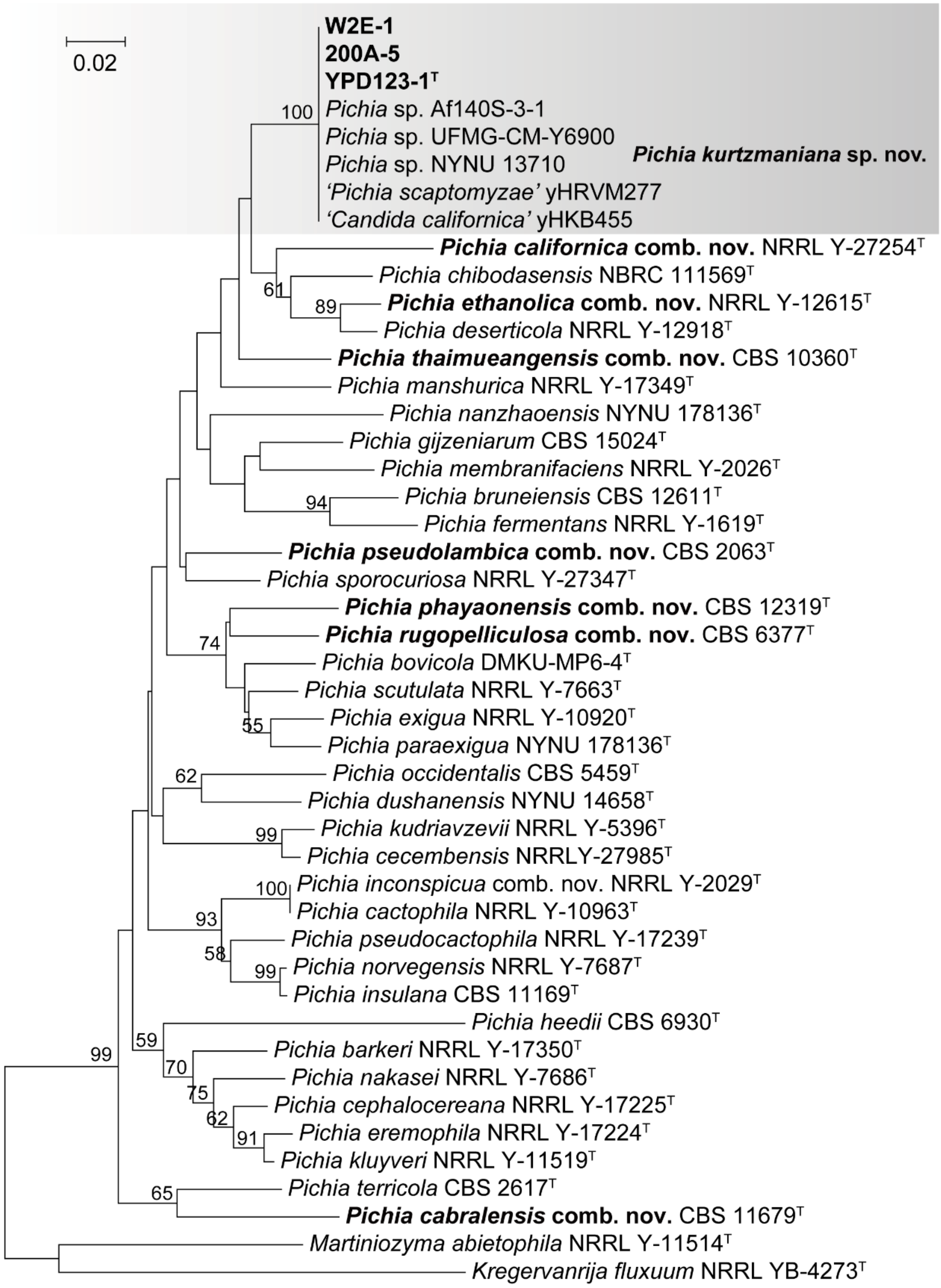

Fig. S2 Neighbor-Joining phylogenetic tree based on the ITS sequences showing the phylogenetic position of *Pichia kurtzmaniana* sp. nov. Bootstrap percentages >50% from 1000 replicates are shown. Species *Kregervanrija fluxuum* and *Martiniozyma abietophila* are used as the outgroup. Type strains are denoted with the superscript 'T'. Strains isolated in this study and the new combinations are marked in bold. Bar, 0.02 substitutions per nucleotide position.
